# Hepatoprotective Effects of Met-enkephalin on Acetaminophen-Induced Liver Lesions in Male CBA Mice

**DOI:** 10.3390/molecules190811833

**Published:** 2014-08-07

**Authors:** Roko Martinić, Hrvoje Šošić, Petra Turčić, Paško Konjevoda, Aleksandra Fučić, Ranko Stojković, Gorana Aralica, Mario Gabričević, Tin Weitner, Nikola Štambuk

**Affiliations:** 1 Department for Clinical Pathophysiology, Clinical Hospital Centre Split, Šoltanska 1, 21000 Split, Croatia; E-Mail: roko.romast@gmail.com; 2 Department of Urology, University Clinical Hospital Centre Zagreb, Kišpatićeva 12, 10000 Zagreb, Croatia; E-Mail: hsosic@gmail.com; 3 Department of Pharmacology, Faculty of Pharmacy and Biochemistry, University of Zagreb, Domagojeva 2, 10000 Zagreb, Croatia; E-Mail: pturcic@pharma.hr; 4 Center for Nuclear Magnetic Resonance, Ruđer Bošković Institute, Bijenička cesta 54, 10002 Zagreb, Croatia; E-Mail: pkonjev@irb.hr; 5 Institute for Medical Research and Occupational Health, Ksaverska cesta 2, 10001 Zagreb, Croatia; E-Mail: afucic@imi.hr; 6 Division of Molecular Medicine, Ruđer Bošković Institute, Bijenička cesta 54, 10002 Zagreb, Croatia; E-Mail: stojkov@irb.hr; 7 University Hospital Dubrava, Avenija Gojka Šuška 6, 10000 Zagreb, Croatia; E-Mail: garalica@kbd.hr; 8 Medical School University of Zagreb, Šalata 3, 10000 Zagreb, Croatia; 9 Department of General and Inorganic Chemistry, Faculty of Pharmacy and Biochemistry, University of Zagreb, Ante Kovačića 1, 10000 Zagreb, Croatia; E-Mails: mariog@pharma.hr (M.G.); tweitner@pharma.hr (T.W.)

**Keywords:** met-enkephalin, hepatoprotection, genotoxicity, antisense peptide, binding, spectroscopy

## Abstract

Recent histopathological investigations in patients with hepatitis suggested possible involvement of Met-enkephalin and its receptors in the pathophysiology of hepatitis. Consequently, we evaluated the potential hepatoprotective effects of this endogenous opioid pentapeptide in the experimental model of acetaminophen induced hepatotoxicity in male CBA mice. Met-enkephalin exhibited strong hepatoprotective effects in a dose of 7.5 mg/kg, which corresponds to the protective dose reported for several different animal disease models. In this group plasma alanine aminotransferase and aspartate aminotransferase enzyme activities, as well as liver necrosis score were significantly reduced in comparison to control animals treated with physiological saline (*p* > 0.01). The specificity of the peptide hepatoprotection was investigated from the standpoint of the receptor and peptide blockade. It was concluded that Met-enkephalin effects on the liver were mediated via δ and ζ opioid receptors. Genotoxic testing of Met-enkephalin confirmed the safety of the peptide.

## 1. Introduction

Met-enkephalin is an endogenous opioid pentapeptide (YGGFM), also named opioid growth factor (OGF) [1,2]. It is the agonist of δ and ζ opioid receptors, and its pharmacological effects could be blocked by the competitive receptor antagonist naltrexone [1,2]. In addition to the biological effects on neurotransmission and neuroimmunomodulation Met-enkephalin also exhibits strong protective effects in different animal disease models, including gastric cytoprotection [1,2,3,4,5,6,7,8]. It is also primary opioid peptide involved in the cell and tissue growth regulation and wound healing [2].

Recent histopathological investigations suggest the involvement of Met-enkephalin and related opioid system receptors in the pathophysiology of hepatitis and hepatoprotection [9,10]. However, the potential hepatoprotection of this well known neuropeptide has not been defined on the standard animal model. Therefore, we investigated the hepatoprotective effects of Met-enkephalin in the experimental model of acetaminophen induced hepatotoxicity in male CBA mice, a useful animal model of hepatoprotection [11,12,13]. We also tested the specificity of Met-enkephalin mediated hepatoprotection under the conditions of the opioid receptor blockade and Met-enkephalin blockade. Naltrexone was used as a receptor antagonist [1,2], and antisense peptide IPPKY as Met-enkephalin antagonist [6].

## 2. Results and Discussion

### 2.1. Hepatoprotective Effects of Met-enkephalin

Protective potential of Met-enkephalin was evaluated using experimental model of acetaminophen induced hepatotoxicity in male CBA mice, an established screening procedure for the evaluation of hepatoprotective compounds [11,12,13]. The model is especially useful for testing substances with potential anti-inflammatory and anti-necrotic properties [14,15,16]. Protective effects were observed by using three criteria: plasma activities of alanine aminotransferase (ALT) and aspartate aminotransferase (AST) enzymes, as well as liver necrosis score [11,12,17,18,19].

**Figure 1:**
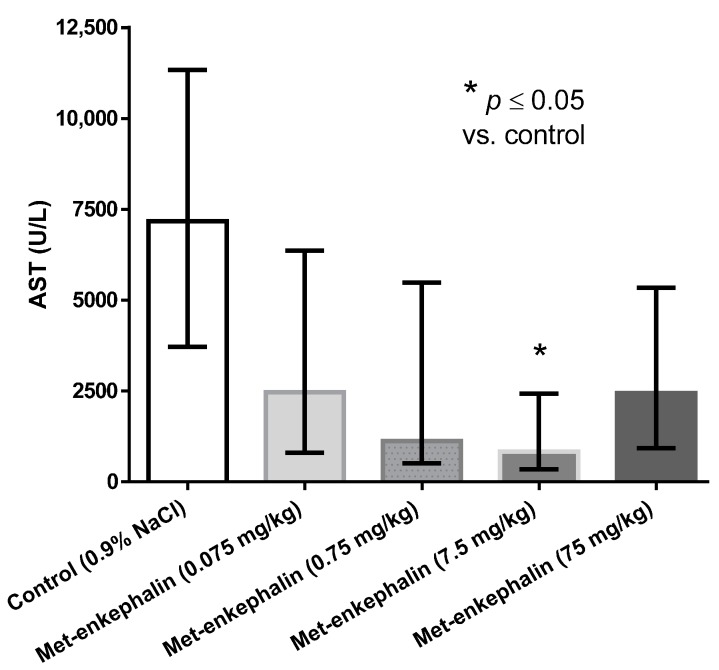
Effects of Met-enkephalin on plasma AST activity (U/L) 24 h after acetaminophen administration. Data are presented as medians and interquartile ranges.

**Figure 2:**
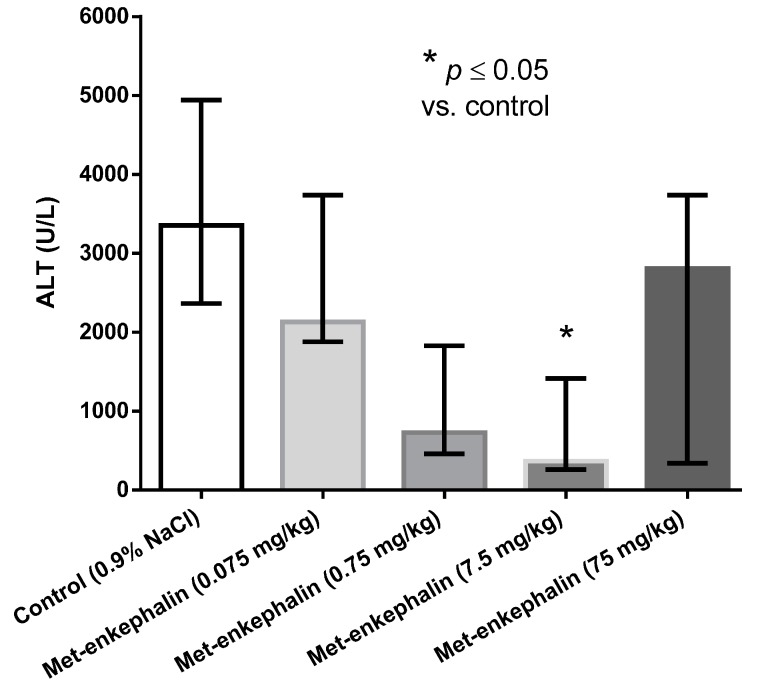
Effects of Met-enkephalin on plasma ALT activity (U/L) 24 h after acetaminophen administration. Data are presented as medians and interquartile ranges.

**Figure 3:**
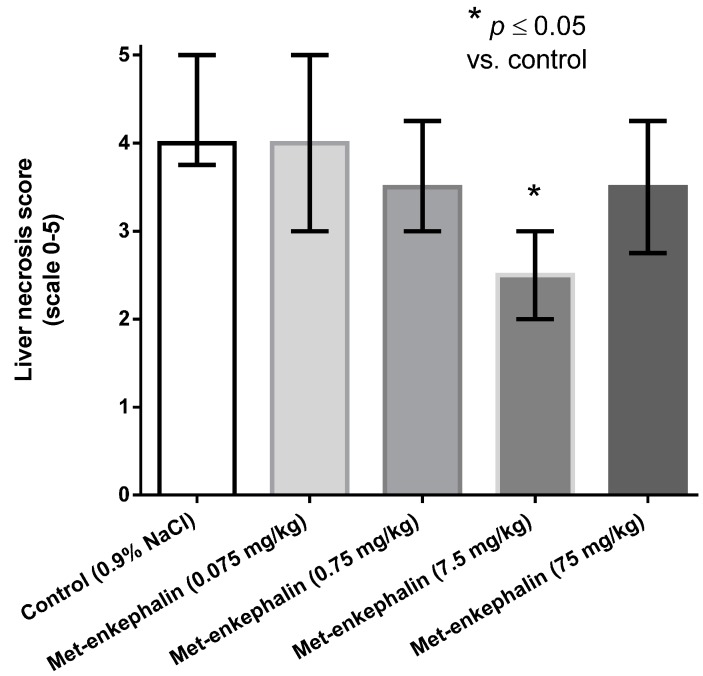
Effects of Met-enkephalin on liver necrosis (scale 0–5) 24 h after acetaminophen administration. Data are presented as medians and interquartile ranges.

Met-enkephalin dose of 7.5 mg/kg was the most efficient dose by all criteria (Table 1, Table 2 and Table 3; Figure 1, Figure 2 and Figure 3). Other doses also showed lowered levels of AST, ALT and liver necrosis in comparison to control animals (0.9% NaCl), however, the results were not statistically significant (Table 1, Table 2 and Table 3; Figure 1, Figure 2 and Figure 3). Figure 1, Figure 2 and Figure 3 suggest U-shaped curve relationship between applied doses and observed effects. This phenomenon is often observed with peptide ligands, including opioid system ligands [20]. The U-shape curve is characterized by low-dose stimulation and high-dose inhibition. However, it is often unrecognized and requires great care in planning and performing the experiments, as well as interpreting the results [20].

The protective dose of Met-enkephalin is in the range of optimal protective doses (4–10 mg/kg) in other animal models of inflammatory/autoimmune diseases in rat, mouse and guinea pig, e.g., experimental allergic encephalomyelitis, histamine induced bronchoconstriction, arthus skin reaction, delayed skin reaction, adjuvant arthritis, allograft rejection, anaphylactic shock [3,4,5,6,7,8].

**Figure 4:**
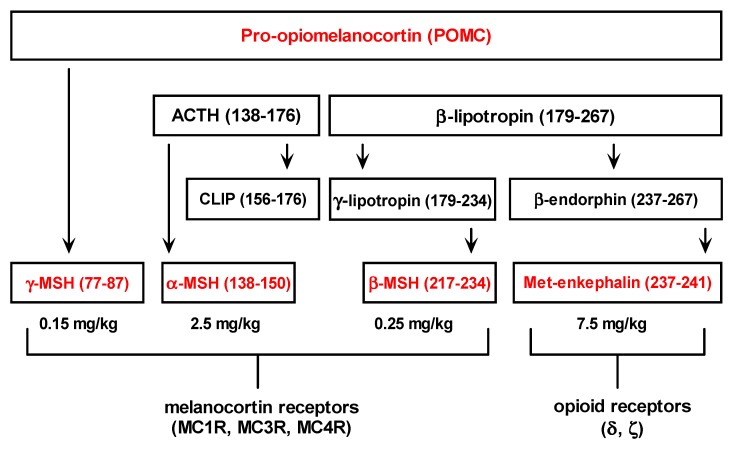
Pro-opiomelanocortin derived peptides and relevant doses that exert their hepatoprotective effects in the experimental model of acetaminophen induced hepatotoxicity in male CBA mice [11,12,13].

Met-enkephalin sequences are found in proenkephalin (PENK) and pro-opiomelanocortin hormones (POMC). It is worth mentioning that other pro-opiomelanocortin (POMC) peptides also exhibit hepatoprotective effects in the same hepatotoxicity model (Figure 4) [11,12,13]. Consequently, opioid and melanocotrin classes of G-protein-coupled receptors seem to be involved in the regulation of liver inflammation [11,12,13,21].

**Table 1:** Effects of Met-enkephalin on AST activity in plasma (U/L) 24 h after acetaminophen administration (150 mg/kg i.g.). Met-enkephalin was given intraperitoneally 1 h before acetaminophen. * comparison with control using Steel’s test.

Group (*n* = 8)	Mean	SD	Median	*p* value *
1. Control (0.9% NaCl)	7545	4114	7170	
2. Met-enkephalin (0.075 mg/kg)	3362	3052	2470	0.2169
3. Met-enkephalin (0.75 mg/kg)	2621	3959	1127	0.0967
4. Met-enkephalin (7.5 mg/kg)	1313	1317	836	0.0039
5. Met-enkephalin (75 mg/kg)	3161	2982	2440	0.1190

**Table 2:** Effects of Met-enkephalin on ALT activity (U/L) in plasma 24 h after acetaminophen administration (150 mg/kg i.g.). Met-enkephalin was given intraperitoneally 1 h before acetaminophen. * comparison with control using Steel’s test.

Group (*n* = 8)	Mean	SD	Median	*p* value *
1. Control (0.9% NaCl)	3946	1752	3355	
2. Met-enkephalin (0.075 mg/kg)	2547	1263	2139	0.7596
3. Met-enkephalin (0.75 mg/kg)	1062	821	730	0.0751
4. Met-enkephalin (7.5 mg/kg)	794	911	370	0.0072
5. Met-enkephalin (75 mg/kg)	2194	1765	2810	0.6620

**Table 3:** Effects of Met-enkephalin on liver necrosis (scale 0–5) 24 h after acetaminophen administration (150 mg/kg i.g.). * comparison with control using Steel’s test.

Group (*n* = 8)	Minimum	Q1	Median	Q3	Maximum	*p* value *
1. Control 1 (0.9% NaCl)	3	3.8	4.0	5.0	5	
2. Met-enkephalin (0.075 mg/kg)	3	3.0	4.0	5.0	5	0.9657
3. Met-enkephalin (0.75 mg/kg)	3	3.0	3.5	4.3	5	0.5495
4. Met-enkephalin (7.5 mg/kg)	2	2.0	2.5	3.0	4	0.0094
5. Met-enkephalin (75 mg/kg)	2	2.8	3.5	4.3	5	0.4770

δ and ζ opioid receptors are present in the liver tissue, however, both opioid receptors subtypes share little sequence homology and have quite different function [2,9,10]. Beneficial effects of δ opioid receptors in the liver could be related to the modulation of immune mediated tissue injury and oxidative stress, while ζ opioid receptors are involved in the regulation of tissue growth and wound repair [1,2]. Consequently, strong hepatoprotection exerted by Met-enkephalin dose of 7.5 mg/kg may result from the peptide effects on both opioid receptor subtypes.

### 2.2. Modulation of Hepatoprotection with Naltrexone and Antisense Peptide

**Figure 5:**
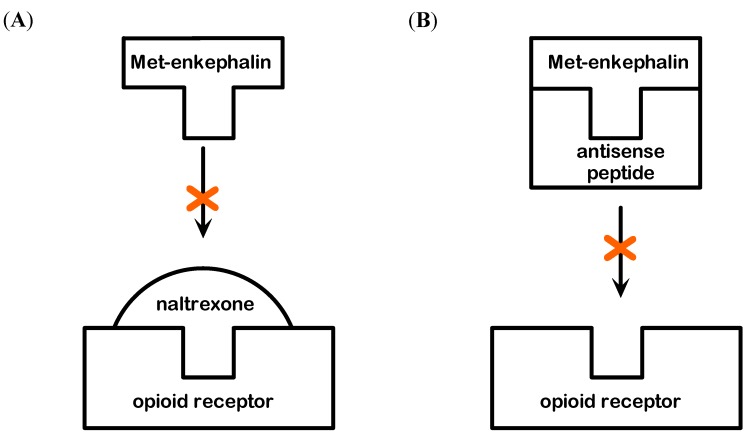
(**A**) Modulation of Met-enkephalin binding to δ and ζ opioid receptor by menas of the opioid receptor antagonist naltrexone. (**B**) Modulation of Met-enkephalin binding to δ and ζ opioid receptor by means of antisense peptide antagonist.

The specificity of Met-enkephalin hepatoprotection was investigated from the standpoint of the receptor and peptide blockade (Figure 5). It is believed that the effects of Met-enkaphalin are mediated via δ and ζ opioid receptors [1,2,4]. The blockade of these receptors with specific antagonist naltrexone confirmed this fact, because the effects of Met-enkephalin were completely abolished, *i.e.*, the mortality rate of this group was high (6/8). Similar mortality was observed when antisense peptide IPPKY was applied with Met-enkephalin (5/8), or alone (4/8). This mortality rate probably results from the blockade of both pharmacologically applied and endogenous Met-enkephalin. Sense-antisense peptide complex could also elicit adverse biological effects [22].

The blockade of Met-enkephalin with naltrexone suggests that the effects are mediated via δ and ζ opioid receptors, while the blockade of Met-enkephalin with antisense peptide [6,13,22,23] allowed us to observe the hepatoprotection/hepatotoxicity in the state of fully preserved receptor function, enabling other endogenous substances to act on them. It can be concluded that Met-enkephalin effects on the liver are peptide specific and mediated via δ and ζ opioid receptors.

### 2.3. Genotoxic Testing of Met-enkephalin

In order to test the genotoxic potency of Met-enkephalinan *in vivo* micronucleus (MN) assay was used, which enables detection of clastogenic and aneugenic mechanisms [24]. Selection of the doses was based on the previously published results, stating that 4–10 mg/kg is the relevant therapeutic Met-enkephalin dose for cytoprotective and organ protective experimental mice models *in vivo* [3,4]. The assay requests small sample and it is possible to use same animals for estimation of background value and for sampling during the experiment, which significantly improves the reliability of results. Erythroblasts, as cell type, are optimal for the investigation of genome damage due to high rate of division and life span in peripheral blood. As erythroblasts circulate through organism they act as natural bio-dosimeters. The limitation of this method is that it is not possible to recognize whether the mechanism by which tested agent may cause genome damage is preferentially aneugenic or clastogenic.

Data were modeled using Poisson distribution and presented as rates (number of MN per 1000 cells/reticulocytes), and their 95 per cent confidence intervals. There was no significant difference between male and female mice. As a positive control cylophospahmide at a concentration of 10 mg/kg caused significant increase in MN frequency (12.62/1000 cells). Exposure of animals to concentrations of Met-enkephalin of 0.5 mg/kg, 5 mg/kg and 50 mg/kg did not increase the MN frequency in comparison with background values both at 48 and 96 h after application. There was no significant changes of MN frequency for concentrations of 0.5 mg/kg, 5 mg/kg and 50 mg/kg between sampling times (Table 4).

Our *in vivo* results in the large range of Met-enkephalin doses on a relevant and well known method to test the genotoxic potency of the substance, confirm previous pre-clinical experiments and clinical trials indicating the safety of the peptide in this respect [1,5,6,25].

**Table 4:** The results of genotoxic testing of Met-enkephalinan using *in vivo* micronucleus assay. * 95 per cent confidence interval.

Group (*n* = 8)	Background Value	Exposed 48 h	Exposed 96 h
	Mean Rate (95% CI) *	Mean Rate(95% CI)*	Mean Rate(95% CI)*
1. Met-enkephalin (0.5 mg/kg)	0.13 (0.05–0.23)	0.24 (0.14–0.37)	0.29 (0.18–0.43)
2. Met-enkephalin (5 mg/kg)	0.13 (0.05–0.23)	0.22 (0.12–0.34)	0.29 (0.18–0.43)
3. Met-enkephalin (50 mg/kg)	0.09 (0.03–0.24)	0.14 (0.07–0.26)	0.09 (0.04–0.18)

## 3. Experimental Section

### 3.1. Test Compounds

1. Met-enkephalin (YGGFM, mw 573.66, >99% purity; Biofactor GmbH, Bad Harzburg, Germany).
2. Antisense peptide (IPPKY, mw 616.75, 98.5% purity, and IPPKYW, mw 802.96, >99% purity; GenScript, Piscataway, NJ, USA).
3. Naltrexone hydrochloride (mw 377.86, >99% purity; Sigma-Aldrich Co. LLC, St. Louis, MO, USA).

### 3.2. Treatment Regimen and Experimental Models

Hepatotoxicity and Genotoxicity experiments were performed according to the ILAR Guide for the Care and Use of Laboratory Animals, Council Directive 86/609/EEC, and Croatian Animal Protection Act (Official Gazette 135/06) [26,27,28]. The experiment on animals was approved by the Croatian Ministry of Agriculture, Forestry and Water Management.

#### 3.2.1. Hepatotoxicity Model

The experimental animals were 12–16 weeks old male CBA mice, weighing 20–25 g, bred at the Ruđer Bošković Institute. The animals were kept in a room with dark-light cycle (12/12 h) and constant temperature (22 ± 1 °C). Hepatotoxicity was induced according to the slightly modified procedure of Guarner *et al.* [29,30]. Mice were given 0.3 g/L phenobarbital (Phenobarbiton Pliva, Zagreb, Croatia) for 7 days, to induce hepatic drug-metabolizing enzymes. Prior to inducing liver damage by acetaminophen the animals were fasted overnight with free access to water. Acetaminophen (Sigma-Aldrich Co.) was given intragastrically (i.g.), in a dose of 150 mg/kg, via a gastric tube, in a volume of 0.5 mL. Mice were re-fed after 4 h. The test substances were given intraperitoneally (i.p.) 1 h before acetaminophen administration, in a volume of 0.2 mL.

We tested: hepatoprotective effects of: (1) Met-enkephalin, (2) antisense peptide IPPKY, (3) naltrexone, (4) equimolar administration of neltrexone and Met-enkephalin (naltrexone was given 30 min prior to Met-enkephalin), and (5) equimolar mixture of Met-enkephalin and antisense peptide (mixed together 30 min prior to administration under the same physicochemical conditions used for spectroscopy binding experiment described in Section 3.3.). Control animals were treated with physiological saline (0.9% NaCl). The size of experimental groups was 8.

The experimental animals were sacrificed 24 h after acetaminophen application. For biochemical analyses 250 IU of heparin was given intraperitoneally (i.p.) to each animal 15 min before sacrifice, and the trunk blood was collected into heparinized tubes. Alanine aminotransferase (ALT) and aspartate aminotransferase (AST) activity was determined on an Olympus AU**^®^** 400 analyzer using standard reagents. Mice that died of acetaminophen toxicity before 24 h period were excluded from the biochemical and/or histopathological analysis [17]. In control and Met-enkephalin treated groups only one experimental animal died of acetaminophen toxicity (Met-enkephalin in dose of 0.75 mg/kg).

For histopathological analysis sections of the liver were fixed in 10% phosphate buffered formalin and embedded in paraffin. Three specimens were taken from each liver, cut into six sections of 3 μm, and stained with hemalaun-eosin (HE). Sections were examined using light microscope (×100). Grading of the liver lesions was done on 0–5 point scale: 0 = no lesions; 1 = minimal lesions (individual necrotic cells); 2 = mild lesions (10% to 25% of necrotic cells or mild diffuse degenerative changes); 3 = moderate lesions (25% to 40% of necrotic cells); 4 = marked lesions (40% to 50% of necrotic cells); and 5 = severe lesions (more than 50% of necrotic cells)) [19]. The final score for each liver was the consensus score of all examined sections.

#### 3.2.2. Genotoxicity Testing

The study included four male and four female (BALB/CJ) mice per each applied compound concentration. The mice were obtained from the Ruđer Bošković Institute (Zagreb, Croatia) breeding colony. During the experiment period four animals were kept per cage. The bottom of cage was covered with sawdust (Allspan^®^, Karlsruhe, Germany). Standard food for laboratory mice (4 RF 21 GLP Mucedola srl, Milan, Italy) was used. All animals had free access to food and water ad libitum. Animals were kept in conventional conditions with the light/dark cycle exchanging every 12 h, temperature 22 °C, and humidity 55%. Positive control was performed by 10 mg/kg of cyclophosphamide (CP) (Krka, Novo Mesto, Slovenia). Met-enkephalin was administered i.p to animals as single dose at concentrations of 0.5 mg/kg, 5 mg/kg and 50 mg/kg. Blood samples were taken from mice before treatment, 48 h and 96 h after treatment. The advantage of this method is a small sample size, which is why the same animals can be used for repeated measurements with diminished possible bias of inter individual variability.

For all groups, peripheral blood (5 µL per sample) was collected from the tail vein. The blood was smeared on an acridine-orange coated slide, covered with a cover slip and analyzed according to Hayashi *et al.* [24]. Micronuclei were analyzed in 2,000 reticulocytes per sample. Data were analysed using pois.exact (epitools package) and poisson.test (exactci package) procedures inside R data analysis software [31,32,33].

### 3.3. Peptide Binding Assay Using Tryptophan Fluorescence Spectroscopy

Antisense peptides specified by the complementary RNAs bind to each other with enhanced specificity and affinity due to the principle of amino acid complementary hydropathy, and consequently may be used to abolish the biologic activity of the sense peptides/hormones [13,22,23,34,35,36]. This biologic phenomenon has been proved for more than 40 peptide-peptide interaction systems, including Met-enkephalin, and represents a useful tool for the investigation of peptide-receptor systems [6,22,23,34,35,36].

**Figure 6:**
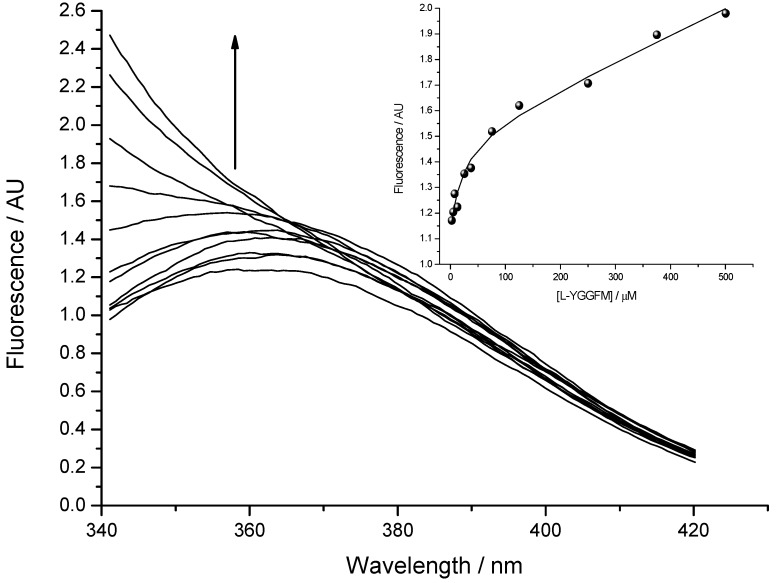
The binding of Met-enkephalin and antisense peptide (K*_d_* = 19 ± 3 µM; mean ± SD) evaluated by means of fluorescence spectroscopy. Fluorescence in arbitrary units (AU) is given as a ratio of signals obtained from sample and reference PMTs. **Inset**: Fitting curve at 350 nm.

Fluorescence spectra were measured by OLIS RSM 1000F spectrofluorimeter (Olis, Inc., Bogart, GA, USA) equipped with thermostated cell at 25 °C [13,23]. The concentration of Met-enkephalin varied from 2.5 to 500 μM. Figure 6 presents titration of 2.5 µM solution of IPPKYW with Met-enkephalin at 25 °C, pH = 7.4, in 10 mM phosphate buffer. Both reactants were fluorophores and the third spectrally active species was attributed to the complex of two reactants. Phenylalanine Met-enkephalin, which was in excess, had much smaller quantum yield than the tryptophan present in IPPKYW. The excitation wavelength at 290 nm was chosen in order to diminish the fluorescence of phenylalanine and maximise the fluorescence of tryptophan. All spectra in fluorescence titrations were analysed with SPECFIT software and three spectrally active species were suggested by SVD (single value decomposition) statistical analysis [37,38,39,40,41]. Data analysis suggested 1 to 1 complex formation and did not indicate any higher order complexes. Consequently, proposed model is given by Equation (1) and Equation (2) where K*_d_* is dissociation constant of the complex:

IPPKYW − YGGFM↔IPPKYW + YGGFM
(1)

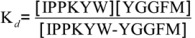
(2)

Dissociation constant (K*_d_*) calculated from fluorescence titrations for the complex of Met-enkephalin with IPPKYW peptide was 19 ± 3 µM (mean ± SD).

### 3.4. Statistical Analysis

Statistical analysis and plotting was made using GraphPad Prism for Windows version 5 and KyPlot version 4 [42,43]. AST and ALT activities are described as means, medians, and standard deviations. Liver necrosis scores are described as minimum, first quartile (Q1), median, third quartile (Q3) and maximum. Data in plots are shown as medians and interquartile range. Differences between the groups were analyzed using Steel’s test. All tests were two-tailed, and the results were considered significant if the *p* values were ≤ 0.05 [44].

## 4. Conclusions

1. Met-enkephalin showed protective effects in the model of acetaminophen induced hepatotoxicity in male CBA mice.
2. The optimal hepatoprotective dose of Met-enkephalin was 7.5 mg/kg, which is in the range of protective doses (4–10 mg/kg) observed in animal models of inflammatory/autoimmune diseases.
3. Met-enkephalin effects on the liver are peptide and receptor specific, mediated via δ and ζ opioid receptors.
4. Genotoxic testing of Met-enkephalin confirmed the safety of the peptide.
